# Painful subjects: the sociogenic processing of pain in individuals with Ehlers-Danlos syndromes

**DOI:** 10.1016/j.ssmmh.2026.100634

**Published:** 2026-04-25

**Authors:** Tom A. Doyle, Samantha L. Vershaw, Colin M.E. Halverson

**Affiliations:** a Center for Bioethics, Indiana University School of Medicine, Indianapolis, IN, USA; b Department of Medicine, Indiana University School of Medicine, Indianapolis, IN, USA; c Department of Anthropology, Indiana University, Indianapolis, IN, USA; d Regenstrief Institute, Indianapolis, IN, USA; e Charles Warren Fairbanks Center for Medical Ethics, Indianapolis, IN, USA

**Keywords:** Chronic pain, Ehlers-Danlos syndromes, Clinician doubt, Subjectification, Social power dynamics, Patient-provider interaction

## Abstract

It is well demonstrated that the chronic pain experienced by individuals with Ehlers-Danlos Syndromes (EDS) has a negative effect on their overall quality of life. Despite this, this population experiences high levels of doubt and invalidation from clinicians regarding the frequency and intensity of their pain. In this qualitative study, we interviewed 39 individuals with EDS in order to investigate how experiencing doubt from clinicians impacted their self-perceptions of pain. Contrary to the claim that pain is a purely private phenomenon, our participants’ experiences were mediated by social dynamics related to epistemic and power asymmetries. These asymmetries affected their willingness to acknowledge their pain to others, to judge it against the experiences of those whom they considered biomedically “normal,” and even to discount the reality of their pain experience. In analyzing these findings, we examine the discursive and intersubjective forces that shape the experience of chronic pain in this population and argue for the expansion of the biopsychosocial model to include these sociogenic processes by which patients rearticulate and reconceptualize their pain to match clinical expectations of “normal” symptoms.

## 1. Introduction

In 1973, a landmark study published in the *Annals of Internal Medicine* raised the concern that chronic pain was being inadequately treated. While the study's authors gestured towards insufficient provider education as the proximate cause, they recognized the difficulties in evaluating pain: They acknowledged that pain is “subjective” and therefore its assessment depends upon self-reporting, which can be influenced by numerous extraneous factors such as personality, cultural background, and a “desire to please the doctor or to mobilize help from him” (Marks and Sachar, 1973). Subsequent scholarship has expressed skepticism that patients can be reliable reporters of their own symptoms, and that such a private phenomenon can be adequately evaluated by external observers (Huskisson, 1974). In response, the American Pain Society (APS) began to hold scientific symposia on the topic (Max, 1995). In 1995, the resulting consensus statement was published in the *Journal of the American Medical Association*, describing persistent deficiencies in pain treatment. The statement offered numerous guidelines, the first of which directed clinicians to “chart and display patients’ self-report of pain” using numeric scales and then record these data “on a vital sign sheet at the patient's bedside” (Max, 1995). This guideline evolved into James Campbell's highly influential assertion that that pain should be treated as the “fifth vital sign” (Campbell, 1996). However, unlike the four traditional vital signs, it was determined that charting pain could not rely on objective measures, codifying the importance of subjectivity in the evaluation of this significant symptom. To this day, the International Association for the Study of Pain indicates that pain must be understood as an irreducibly subjective experience (Raja et al., 2020).

However, recent studies have raised skepticism about the effectiveness of treating pain as one does the other vital signs (Scher et al., 2018; Lucas et al., 2007; Ballantyne and Sullivan, 2015), and an emerging body of evidence points to certain pain scales’ being largely ineffective at addressing patient's pain (Halverson and Doyle, 2023; Gordon, 2015; Mularski et al., 2006). In one troubling instance, a study found that the implementation of a numeric pain scale at a hospital correlated with higher instances of opioid-related adverse drug reactions (Vila et al., 2005). Therefore, some scholars contend that progress in symptom management demands an account of pain that describes how such a subjective experience can be influenced not merely by an individual's biological but also psychological and social contexts (Fillingim, 2017). This biopsychosocial model theorizes that pain can be exacerbated by a wide range of trigger classes (Waddell et al., 1984; Loeser et al., 1982). For this reason, addressing the multifaceted nature of this symptom requires attention not just to its physiological factors (i.e., noxious stimuli and neurochemical responses) but to its psychological (e.g., catastrophizing, depressive feelings, anxieties) and social factors (e.g., stigmatization and alienation) as well (George et al., 2012; Nicholas, 2022).

In the current study, we advance this line of theorizing. While in many ways, it is conceptually useful to understand pain as a private or subjective experience, we consider how it is additionally influenced by epistemic and power dynamics. More specifically, we investigate pain as, in part, discursively and intersubjectively produced. In this way, we add complexity to the prevailing theoretical description of pain as “original firsthand experience” (Geniusas, 2020).

Our investigation was motivated by previous work conducted among individuals living with Ehlers-Danlos Syndrome (EDS), an uncommon hereditary disorder of connective tissue. There are currently thirteen named types of EDS, not including the related Hypermobility Spectrum Disorder. Twelve of these types have a known genetic component, for which reason we refer to them collectively as the molecularly defined types (mdEDS). The remaining type – diagnosed solely based on clinical criteria – is by far the most common, and is called hypermobile EDS (hEDS) due to its characteristic joint hypermobility as assessed by a Beighton score. Unless specified, instances of “EDS” in this article refer to the collective concerns of both individuals with mdEDS and those with hEDS.

Over the last two decades, it has been increasingly acknowledged that EDS manifests in large part as a chronic pain syndrome (Camerota et al., 2011; Castori et al., 2013; Sacheti et al., 1997; Voermans et al., 2010; Zhou et al., 2018). For this reason it is sometimes referred to as an invisible illness, whose subjective experience cannot easily be measured using objective medical tools (Sacheti et al., 1997; Voermans et al., 2010; Scheper et al., 2015). Qualitative studies have demonstrated that individuals with EDS, like many similar populations (e.g., individuals with lupus, fibromyalgia, or rheumatoid arthritis), often encounter doubt and invalidation from their clinicians (Halverson et al., 2021; Berglund et al., 2010). Their chronic musculoskeletal pain, which may express itself in unanticipated and heterogeneous ways, is frequently scrutinized, downplayed, and dismissed (Berglund et al., 2010; Trudgian and Flood, 2024; Hill et al., 2025). Such external doubt has been found to transmute into a patient's internalized self-doubt about the reality of their experiences as well as about the overall validity of EDS as a diagnostic category (Halverson et al., 2023a). The current paper is our attempt at broadening and sharpening the way we model chronic pain to capture our participants’ experiences with greater nuance and fidelity.

Informed by phenomenology and post-colonial theory, we developed a theoretical framework to situate our qualitative data set and expand upon how subjective symptoms are shaped by power dynamics. This framework evaluates how interpersonal factors – including power dynamics within the patient–provider relationship – shape private pain experiences. Our framework illuminates how the process of subjectification, as described in the works of Althusser (2008) and Foucault (Dreyfus et al., 1983), influences the phenomenological experience of the subject, as elucidated, for instance, in the works of Husserl (Husserl and Welton, 1999; Zahavi, 2003). We ultimately employ Fanon's concept of sociogeny (Fanon, 2002) to account for how interpersonal factors shape one's inner world, inextricably binding it in a co-constitutive dialectic of cause, effect, and magnification. This sociogenic account of pain is attentive both to the biopsychosocial and to the discursive features of our patients’ experiences.

## 2. Theoretical framework

### 2.1. Two senses of subjectivity

While the term “subjective” has multiple meanings, the theoretical scope of this article focuses on two particular senses. The first pertains to subjectivity defined as private experience, a meaning often associated with phenomenologists who, beginning with Husserl (Husserl and Welton, 1999; Zahavi, 2003), used the term as a descriptor for characteristics of an individual's consciousness. In this sense, subjectivity reflects an individual's possession of a private perspective on the world which cannot be directly shared with others due to the irrevocably distinct nature of conscious experiences. Phenomenologists often attribute the distinction of irrevocability to the fact that we attend to the world as and through physically distinct bodies. While two individuals may possess a *similar* embodiment, they cannot properly be said to possess the *same* body and so must encounter the world in an individualized manner. In the clinical setting, this sense of subjectivity appears in the typification of “subjective symptoms” to denote inherently private experiences, such as dyspnea (i.e., shortness of breath or air hunger), fatigue, and pain.

The second sense of subjectivity characterizes it as the product of dynamic social and institutional processes, captured in part by what Althusser refers to as “interpellation” and what Foucault refers to as “subjectification” (Althusser, 2008; Dreyfus et al., 1983). According to Althusser, state apparatuses (e.g., schools, political parties, families, healthcare systems) “hail” individuals, calling them to inhabit particular ideological roles. Once interpellated, the individuals come to see themselves within that ideology, as the kind of person thus hailed, and comport themselves according to the tenets appropriate to that role. In the case of EDS, for example, a clinician may be said to “hail” individuals, recognizing them as patients – or specific types of subjects – within the ideology of biomedicine. These patients must comport themselves in a manner that befits this role in order for them to appear legible and credible within the healthcare system. If their bodies and concerns fail to conform to biomedicine's expectations, they risk becoming anomalous and ceasing to be treated as legitimate beneficiaries of this state apparatus.

Foucault's concept of subjectification differs from Althusser's in its emphasis on the historical and cultural regimes of power and knowledge that constitute subjects. Foucault shows how the “medical gaze” moves illness from the province of the individual into medicine's jurisdiction (Foucault and Foucault, 1994). Through this process, “human beings are made subjects” within specific fields of power relations (Dreyfus et al., 1983). For Foucault, social institutions like biomedicine enact their power upon individuals: constraining them and making things more or less possible for them by defining what is knowable, sayable, and experienceable for them. Hence, this second sense of subjectivity refers to the ways in which societal forces impinge upon individuals’ self-understanding as subjects within systems of power and knowledge.

### 2.2. Masking

In this article, we put both Louis Althusser's and Michel Foucault's conceptualizations of subjectivity in conversation with one another through the analytic lens of phenomenology and the concept and practice of masking as explicated by Frantz Fanon. We discuss how the processes of subjectification caused our participants to develop and wear “masks” which then influenced attitudes towards certain aspects of their own embodied experiences (e.g., symptoms). Scholars as diverse as Martin Heidegger, Jean-Paul Sartre, and Erving Goffman have argued that an individual's selfhood is not just the product of self-determination, but also a product of the social milieux in which they find themselves. These scholars argue that by encountering the perspectives of others and the broader norms of a society, individuals comport themselves to a particular role within a social setting. Importantly, this comportment affects how an individual experiences themselves; the attitudes, opinions, and beliefs that they hold about themselves are the result of social processing.

However, In order to focus specifically on the power dynamics within the clinical setting, what is needed is an understanding of how certain masks are created and worn by patients in order for clinicians to legitimate their suffering. Such a discussion of how power dynamics influence conceptions of the self and the social presentation of oneself can be found in Fanon's treatment of masking in *Black Skin, White Masks*. Drawing on aspects of existentialism, anthropology, and psychology, Fanon explores how the practice of subjugation influences individuals’ abilities to present themselves within a society. This, in turn, has an effect on how they conceptualize themselves as individuals in private (e. g., in their self-talk or their self-perception). This entire process is what Fanon refers to as sociogeny, how social factors affect the development of the self. Masking plays a key role in the sociogenic process insofar as the creation of these metaphorical masks represents one's subordinate role in a social setting, and the wearing of such masks represents the necessity of mimicking authority in order to retain social legitimacy.

This theory of masking offered by Fanon helps situate our qualitative findings. As we will show, our participants wore masks to match their clinician's expectations and ensure that their pain was treated as legitimate and worthy of clinical attention and treatment. These clinical expectations established what experiences count as pain, often putting participants in conflict with their own embodied experience and self-knowledge. As we will show, our participant's masking impacted their own perceptions and attitudes towards their experiences. In phenomenological terms, their pain *appeared* differently to them as a result of the masking. Thus, we contend that this masking reflects a sociogenic processing of pain.

It is important to note that while masking had an impact on many aspects of our participants’ lives and lived experiences, our analytical and theoretical focus in this paper is on masking with respect to the pain experiences related to EDS. Our use of phenomenological methods is meant to denote only how certain aspects of lived experience (e.g., pain and other symptoms) appear to our participants. Our approach seeks to understand how imbalances of power in the clinical setting result in a sociogenic processing of pain as exemplified through the various masking practices described by our participants.

## 3. Methods

### 3.1. Participants and procedures

Our study team consists of three researchers. One is a male bioethicist with doctoral-level training in qualitative research methods and experience in philosophy of medicine (TAD). The second is a male medical anthropologist and bioethicist with advanced training in qualitative research (CMEH). The third is a female research assistant specializing in improving care for populations living with rare diseases (SLV).

Participants were recruited through three channels: from clinicians specializing in EDS care, who encouraged patients to contact the study team; through flyers distributed through the Ehlers-Danlos Society; and from Annabelle's Challenge, a UK-based charity and advocacy organization.

Participants were required to have a diagnosis of EDS, be 18 years or older, and be proficient in English. They were first invited to complete a pre-survey. Interviewees were then selected from the pool of pre-survey respondents, with a goal of recruiting equal numbers with both hEDS and mdEDS but sampling was otherwise independent of pre-survey responses. Consistent with qualitative research practices, sample size was not predetermined (Saunders et al., 2018). Recruitment continued until the depth and breadth of the collected data was sufficient to develop robust themes related to the experience of doubt—from providers as well as from family and friends—and self-doubt. Interviews were conducted from June to October 2024.

### 3.2. Study design

The pre-survey was designed to gather insights into participants’ experiences of doubt within the healthcare system. It was developed following an extensive review of the literature that include first identifying and reading previous systematic reviews on the topic of clinician doubt and then conducting a backward citation search on relevant articles cited by these reviews. The survey included questions regarding participants' definitions of doubt as a general phenomenon, their communication of pain, attitudes toward pain perception and self-doubt, and experiences of EDS symptoms’ being misclassified as psychogenic.

The interview guide was structured to explore participants' experiences with doubt and its consequences. It was informed by both the literature review and insights from the pre-survey, focusing on key themes identified in prior research regarding doubt in rare disease communities (Halverson et al., 2021, 2023a; Bontempo et al., 2025). Many of the pre-survey questions were revisited to clarify and elaborate on responses, providing more context and self-perceptions of causality. The interview questions were open-ended, and the interviews followed a semi-structured format. Thus, participants were allowed to interpret how best to answer the questions posed to them as well as offer additional insights that they believed were important to the topic of doubt. As a result, interviews generally explored how participants define and relate to doubt and trust, their emotional and cognitive reactions to doubting pain (both in themselves and others), their ability to communicate pain to healthcare providers and loved ones, the impact of doubt on daily life, and how their pain is validated in the face of doubt. Both instruments are available upon request. Participants received a $25 Amazon gift card as an incentive for completing the interview.

### 3.3. Instruments and data analysis

All interviews were conducted by phone or over Zoom with a single researcher (CMEH). Participants were able to complete interviews in a setting where they felt most comfortable, while CMEH conducted the interviews from a private office to ensure confidentiality. Field notes were taken during the conversations, and they were audio-recorded and transcribed using MacWhisper, a HIPAA-compliant AI transcription tool.

The research team then reviewed the transcripts to become familiar with the data and identify key themes. An initial coding tree was developed and refined through team discussion. Transcripts were uploaded to Dedoose, a mixed-methods analysis platform. Reflexive thematic analysis, following the iterative and flexible process outlined by Braun and Clarke was used to analyze the interview data (Braun and Clarke, 2006). This approach allowed the team to identify recurring patterns and relevant themes across interviews, gaining an intimate perspective on the participants’ experiences and stories. Weekly meetings were held to refine the coding tree and ensure consistent use of codes throughout analysis. Themes were then developed through reflection on the codes and coding process, and they were collaboratively defined and reviewed. The results were then narrativized and compiled into a comprehensive report.

### 3.4. Ethical considerations

This research was approved by the Indiana University Institutional Review Board, and all participants provided written and verbal informed consent before enrolling in the study.

## 4. Results

For ease of reference, the results described in detail below have also been briefly summarized in Table 1.

### 4.1. Sample description

Pre-surveys were completed by 43 individuals, 39 of whom went on to complete an interview. Of the interviewees, five different EDS types were represented. Twenty-one had a diagnosis of hEDS (54%) and 18 had been diagnosed with one of four rare, mdEDS types (46%).

The average interview length was 66 min, with a range of 36 to 104 min. The average age of participants was 42, with a range of 21 to 68 years. The majority identified as cis-female (74%) and non-Hispanic white (85%). Approximately three-quarters (74%) had attained a bachelor's degree or higher. Other participant demographics are summarized in Table 2.

### 4.2. Professing pain

#### 4.2.1. Pain–Rejection feedback loop

**Fig. 1.**
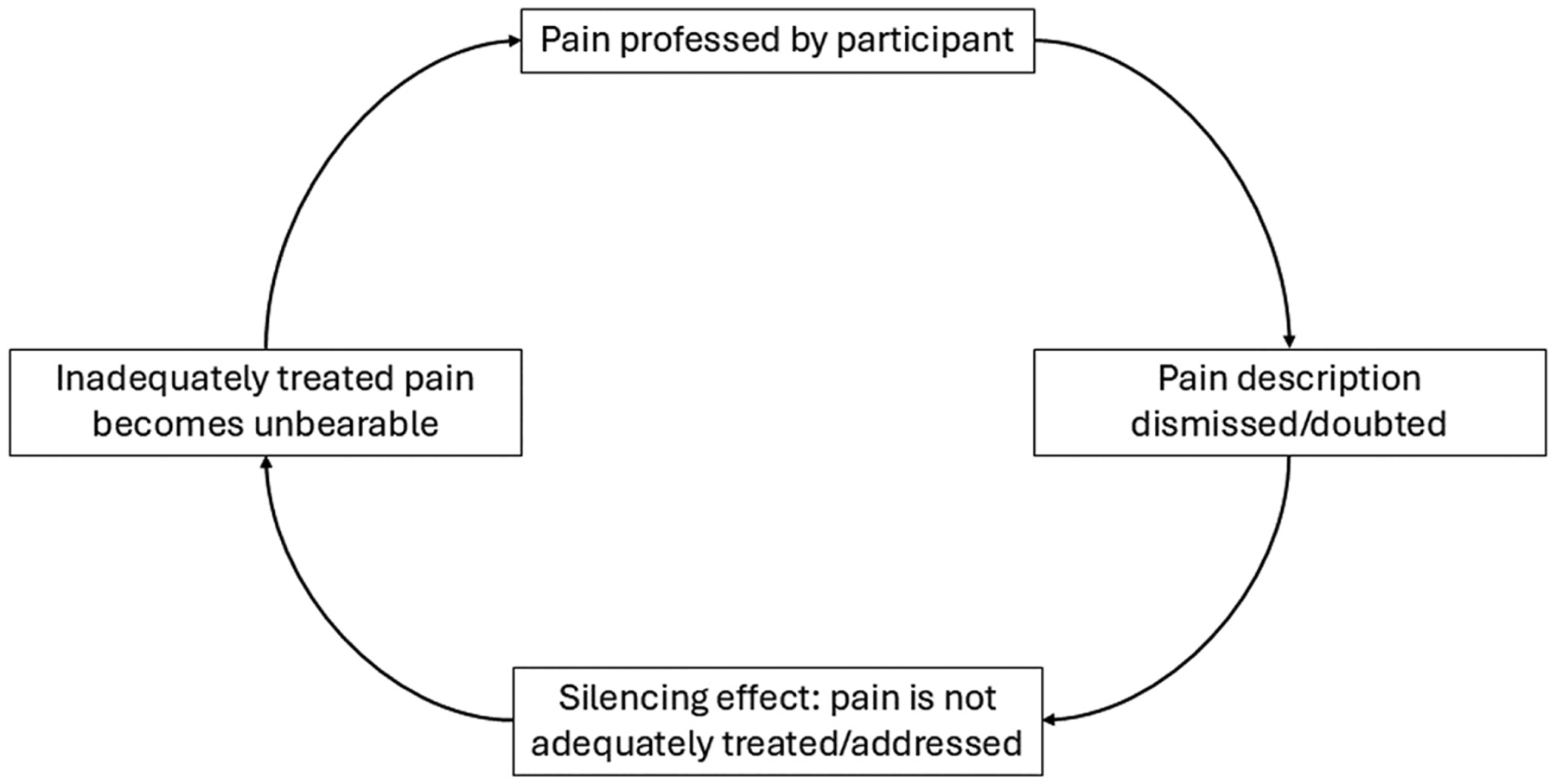
Pain-rejection feedback loop.

About half of our participants described how their clinicians employed their authority as medical professionals to doubt their pain. This dismissal caused a pain-rejection feedback loop (Fig. 1) in which clinicians’ skepticism had a silencing effect, decreasing the likelihood that our participants would describe their pain to others: “If I detect that somebody's doubting me, I shut down and just kind of pass it off, more like: ‘Yep, you're right, everything's fine.’” (831). Although a majority of our participants often believed silence to be the most appropriate response to an authority figure's doubt, over time, this persistent silence resulted in their pain remaining unmanaged and worsening. This, in turn, led to a sense of doubt among our participants that their pain was treatable: “The PCP can't help. The ER doctor can't help. So, who can help?” (328). Eventually, a lack of treatment or inadequate pain management pushed participants to a breaking point, where their pain became unbearable and required them to profess it despite the perceived risks and lack of benefit: “I didn't want them to think I was exaggerating. I wasn't. I was bawling and I couldn't even get out of bed. But I think I let it go for like 48 hours before presenting to the emergency room” (274).

Within this feedback loop, our participants expressed self-doubt occurring immediately after the dismissal of their pain, which then contributed to the silencing stage of the loop. This self-doubt persisted even when participants’ pain became unbearable, with some participants doubting that even severe symptoms warranted treatment (see section 4.4.2).

Notably, participants who encountered a clinician who validated their pain reported experiencing less self-doubt after such encounters. That is, pain validation by clinicians had the opposite effect of pain dismissal: it led participants to be more open about their pain experience rather than silencing themselves. In one instance, a participant described how a clinician's validation of her pain “changed [her] life” (366) because she felt she could be more honest in sharing her pain experiences. Unfortunately, such validating encounters were only experienced by a minority of our participants.

#### 4.2.2. Anticipating dismissal

In some cases, the silencing effect of the pain-rejection feedback loop resulted from our participants mere anticipation of their clinicians’ future dismissal of their symptoms. That is, our participants often anticipated that clinicians would doubt them if they described their pain in a manner that was true to their experience. This reflects the preemptive silencing of their authentic voice in the pain-rejection feedback loop. Participants gave varying reasons for this assumption. In some cases, a participant's worry pertained to the frequency or chronicity of their pain, which they believed would not be congruent with a clinician's expectations: “It would be easy for a clinician to say, ‘Oh, you're overreacting because you're always in pain.’ I think that's why I underplay it, because I don't want to be dismissed” (963). Others relayed a worry that clinicians would doubt the severity of their pain: “I think, overall, I would say that I probably minimize [my pain] because I don't think they'll believe how extreme it really is” (095). In some cases, the concern was more generalized and related to the feeling that they did not fit a conventional pain profile: “I would never talk about [my pain] to literally anyone else, ever […] I think there were some fears of, like, are people just not going to believe [my experience] because it's so different?” (321).

Our participants also described feeling they needed to employ certain strategies in order to circumvent a clinician's dismissal of their pain. In particular, the majority described adapting to what they perceived as the communicative norms of the clinic in order to validate their clinician's epistemic authority. “You say, ‘It's really stiff here’, or things like that, rather than just straight up saying, ‘it hurts so much’, because [… doctors] just shut down after that; they just don't want to hear you. Whereas if you use *their* language, they listen.” (427). One participant felt it necessary to profess their pain in a particular manner that was alienating but aligned better with predetermined social expectations of how pain ought to be expressed: “[The nurse] is like, ‘well, you look fine’. And I'm like, ‘I'm not fine’. So, I'm going back and forth with her. And then I started crying and I'm in hysterics. That's when she believed me […] They won't believe somebody until they're in tears and crying” (108). “That's just dumb,” this interviewee concluded in frustration.

#### 4.2.3. The clinical relevance of pain

As we have seen, disclosing one's pain was viewed as potentially dangerous, threatening social relations and support or causing the individual to appear incredulous to clinicians on whom they depend on for treatment. This culminated in a silencing effect: our participants anticipated their pain would be dismissed by clinicians. However, we also observed that an effect of such dismissal, whether anticipated or actual, led our participants to question whether and when their pain was clinically significant. Asked repeatedly to report their pain levels at clinic visits, the large majority of interviewees struggled to determine when the medical significance of their symptoms was great enough to warrant the risks they faced from disclosure. One participant indicated that she only shared pain that she believed needed immediate medical attention: “If I don't want them to do anything about it, I'm just not going to tell them […] which, I guess, can be a slippery slope in thinking your pain isn't important” (327). Another participant worried that frequently expressing his pain caused others to dismiss it as quotidian. He stated that he did not share his pain, “because I need them to take me seriously when I know it's serious” (408). It was also common for our participants to categorize their pain as either normal or abnormal and then use that categorization in determining whether it warranted the risks associated with disclosure: “I've started answering, ‘nothing beyond the normal’. And then [the clinician] just kind of moves on” from the pain assessment.

### 4.3. Normality and pain

#### 4.3.1. The “Normal” other

It was common for our participants initially to experience their pain as unremarkable, in the sense of both existing below the threshold of concern and of not spending much time commenting on it to others. For the majority of participants, it only occurred to them as abnormal when they began to compare it to the pain experiences of others who they believed experienced or responded to it in a “normal” manner. They assessed their symptoms by witnessing others, taking others’ behaviors and attitudes as representing typical or even healthy qualities and quantities of pain. This comparison led them to understand their pain, or their response to it, as categorically *ab*-normal. Their assessment was not arrived at through self-reflection, but rather through social engagement with “normal” others. For example, one of our interviewees initially spent little time considering the unusual nature of her chronic pain. However, one day she went on a hiking trip through a hilly region, where she saw “people twice my age going right up.” She, on the other hand, struggled due to “feeling like there was glass in my hip” (747). After this excursion, she began questioning the normality of her experiences: “I would be like: ‘Does it really feel like this?’ I mean, everybody else is walking up this hill” (747).

One participant experienced painful subluxations (i.e., the partial dislocation of a joint) while taking part in everyday activities. She grew frustrated and worried when comparing her abilities to those of her colleagues, who could accomplish the same tasks without distress: “Can I just not tolerate normal life, what everyone else is feeling?” (831). Another participant shared that they and a friend underwent the same spinal surgery, but while their friend described their subsequent pain as the “single worst pain ever,” our interviewee found it to be “the easiest thing ever” as it was “only like a fifth of what I deal with every day” (321). They likewise began to reflect on their own pain tolerance in a new light, rooted in the witnessing of others’ experiences.

#### 4.3.2. Discovering abnormality

For close to all participants, chronic pain was a baseline or background noise that they did not initially recognize as problematic or atypical. It only emerged for them as abnormal when identified as such by others; only then did they begin to experience it in this way. These participants began to assess their pain through others’ evaluative frameworks, understanding their experiences as abnormal not through an introspective analysis but through social processing. In one instance, a participant's physical therapist informed him that needing multiple days to recover from exercise was atypical. Our interviewee said this statement “blew my mind, because I was like, ‘That's not normal?’ I thought that was just how everybody works” (328). Another participant similarly described a similar epiphany after a sleep study. He was told that he moved his legs in his sleep “substantially more than anybody they've ever seen before,” which he called “a reaction to constant pain” (688). It was only then, he stated, that he realized the true depth of his pain.

In some cases, this recognition came early in life, during very formative years of psychosocial development. One participant recalled repeatedly being told that she was overreacting. “From a young age I was always told I was making a fuss, or I was making drama, or it couldn't possibly be that bad” (963). This dismissal made her feel isolated and stigmatized: “You grow up thinking everyone else is okay and maybe it’s just me” (963). She said this left her feeling constitutively abnormal, a feeling she has continued to struggle with throughout her life.

#### 4.3.3. Abnormal in a “Normal” world

Another way our participants came to recognize their pain as abnormal was in encountering medical infrastructure and instruments that were designed for able-bodied individuals. For instance, the majority expressed frustration when required to use numeric pain scales, which they found fundamentally ill-suited to reporting their chronic pain: “I think [pain scales are] dumb, because […] somebody who's always in pain has a higher pain tolerance, so when they say it's a seven, somebody looks at them like they're crazy” (095). These scales could not accurately capture our patients’ experiences: “Me having a five is probably you having an 11, you know, because I live with this level every day. And that's where I really struggle to communicate accurately” (301).

Many participants also characterized their pain as ineffable as others do not share the same experiences of background knowledge to interpret their language accurately: “It's very hard to communicate [my back pain] properly because I don't know how it is to not have back and neck pain. So, sometimes I wouldn't even share some information because I didn't know that it was special or different from how other people experience it” (239).

For this reason, an overwhelming majority of participants began to alter the way they used the pain scales, attempting to conform to what they saw as the clinicians’ expectations of their use despite the alienating effects it had on them. Some of this acquiescence was done strategically, so that they would not appear to be bad historians, malingering, or exaggerating. But for others, it was the result of internalizing the clinician's numeric rating as the true, more accurate assessment of their perceptions. For instance, one man noted that following a retinal detachment, he initially believed he was experiencing “a ten out of ten”. However, his clinician misidentified the source of his extreme pain and said the severity must be much lower. This assertion reportedly led our participant to reevaluate his initial assessment, coming to believe his pain was in fact “a four or five” (271).

### 4.4. The self-assessment of pain

#### 4.4.1. Is the pain real?

Throughout our interviews, the majority of participants shared that clinicians’ doubt regarding their description or reaction to pain, led them to question whether their symptoms were real. “For a long time, I started to think I was crazy. Like, I must be making [my pain] up myself” (199). Another participant similarly shared her experiences in clinic, stating that “nobody wants to acknowledge [the pain] or do anything about it or act like it's a problem that should be treated, countless times, telling physician after physician: ‘I'm in a lot of pain’ and they kind of are like, ‘Okay,’ and that's it. It makes you feel like maybe you're just making it all up” (831). One participant reported that she was misdiagnosed by a clinician as having functional neurological disorder (FND) after she repeatedly described her symptoms to him. FND is a neuropsychiatric processing disorder in which sensations such as pain, or events such as seizures or sudden falls, are triggered by a dysfunction in the function of the brain, rather than a dysfunction of its structure. In response to this FND misdiagnosis, she began to question the reality of her first-personal experiences: “Why do I experience all this pain, then? Does that mean the pain never existed?” (648).

A small subset of our participants described internalizing the doubt encountered by clinicians whom they respected as medical authorities. Over time, they started to see themselves through the same skeptical lens. They often questioned the reality of their pain in an enduring and deep-seated manner, so that even when no one else was present to question them, they adopted this iatrogenic doubt. As one participant put it, “I tend to doubt a lot, if what I'm feeling is what I'm actually feeling or if I'm making it up, even though I'm not even sharing it with someone” (239). In some cases, clinicians’ repeated doubt reportedly caused our participants to believe that they might not have EDS at all. Paradoxically, when therapies temporarily relieved their chronic pain, some participants took this as legitimizing their clinician's doubt and began questioning their ability to assess their own experiences.

#### 4.4.2. Is the pain really That bad?

We also found that a minority of participants struggled to assess the severity of their pain after others downplayed or minimized its significance. One participant explained how the experience was similar to having a “voice in your head, telling you the things that other people have told you before, like, ‘it can't be that bad’” (239). In one instance, a woman shared how her friends had told her that she was just clumsy, and this label had led her to believe that her pain was less significant and less worrisome than it really was: “I just thought I was just a klutz. I just get hurt all the time” (108). Learning that others would not believe the severity of their pain, they downplayed it: “Will they believe you when you tell them how bad it is?” (327).

A small group of participants found value in this internalized doubt as well, coopting it as a strategy to avoid the medically worrisome implications of their pain. They did not want to believe it: “I do downplay [my pain] to myself. It's a fear of what it might be. […] It's a fear of: What if they find something?” (963). Several participants mentioned indulging others’ skepticism as a tactic to push themselves to engage in activities they otherwise would consider too dangerous. They magnified the alienating voice of the other that they had internalized and convinced themselves of the appropriateness of downplaying their pain.

#### 4.4.3. The validity of seeking care

Beyond disclosing their pain to others, the majority of participants struggled internally over whether their pain was legitimate or severe enough to warrant medical attention. Experiencing clinicians’ doubt led them to question not merely whether they would be believed in the moment, but whether they were actually capable of interpreting their private experiences accurately: Could they discern whether something was a symptom or a mundane sensation? One participant used to ask herself whether she was “just being silly,” or whether she “jumped the gun a bit” when seeking pain management in clinic (255). Even after discovering that a certain pain in her leg was the result of multiple aneurysms likely caused by her vEDS, she nevertheless continued to worry that she had wasted her clinicians’ time. Another participant avoided seeking treatment for her pain because she did not “want to waste people's time” in the off chance that it was not as severe as she believed (223). Going to the hospital felt to her like an unwarranted imposition on the clinicians. Another interviewee stated: “I've gotten to the point where I'm like, ‘This is severe’, but my ability to judge what is dangerous is completely compromised […] my threshold for understanding when it is dangerous is completely gone” (791).

## 5. Discussion

### 5.1. variability in pain experiences

Although numerous articles have indicated that obtaining a genetic diagnosis may make a substantial improvement in a patient's overall care (Marshall et al., 2019; Splinter et al., 2018; Klitzman et al., 2024), we were unable to find any meaningful differences in how our participants with hEDS and mdEDS experienced clinicians’ doubt or the frequency with which they experienced these doubts. Additionally, we did not uncover any meaningful gender-based variability in the pain experience or clinical doubts reported. However, some female participants gestured to their overall clinical mistreatment as being motivated by sexism and misogyny. Other studies have been able to establish an association between female gender identity and increased rates of pain dismissal and clinical mistreatment (Igler et al., 2017; Brown et al., 2024; Hoffmann and Tarzian, 2001; Cleghorn, 2022). Finally, some participants reported that they had less self-doubt about their pain as they had gotten older, suggesting that age may be another demographic feature relevant to the incidence and experience of doubt and self-doubt.

### 5.2. Pain's social dimension

It is unequivocal that pain is, in an important sense, a private experience; if someone were to dislocate their shoulder, only that person would experience the pain that arises from this personal injury. Nonetheless, our study reveals that these private experiences are socially and discursively mediated, shaping their interpretation and even perception of their symptoms. Power asymmetries present in the patient–provider relationship influence patients’ attitudes and beliefs regarding their pain experiences. Significantly, we found that doubt and self-doubt are reflexes of the power and knowledge imbalance between patient and provider. In many cases, our participants interpreted clinicians’ doubt as suggesting that they lacked the necessary expertise to verify, assess, and address their subjective experience, a stance they then internalized. The act of doubting, then, functioned not merely as an interpersonal evaluation but a sociogenic force formatively influencing their private pain.

Anthropologists have argued that pain is neither purely private nor purely biological, but rather have stressed its relational and sociocultural dimensions. Jean Jackson, for instance, notes that “although biomedically and conventionally pain is seen as a property of an individual, it is in fact deeply intersubjective” and mediated through social relations and shared meaning (Jackson, 2011). Likewise, Veena Das (Das et al., 2010), Talal Asad (Asad, 2011), and others have shown that pain is inseparable from the politics and power of recognition. It can be a plea for acknowledgement and a means of resisting “menacing societal pressures” (Kleinman, 1997). Anthropological critiques of so-called Cartesian dualism further underscore that pain always emerges from and is embedded in the patient's sociohistorical environment (Aydede and Güzeldere, 2002).

Much of this literature, however, focuses on the social meaning of pain, or on public pain behavior, which Ravinder Singh argues is the voluntary and culturally conditioned dimension of pain expression (Singh, 2017). Our data complement these accounts by illustrating how sociocultural forces penetrate more deeply, shaping not only the interpretation or expression of pain but its phenomenological experience: what patients feel, how they attend to their sensations, and whether they regard those sensations as real.

Our findings indicate that the expression and – more fundamentally – experience of pain is modified by the doubt that individuals encounter from others, especially their clinicians. Those suffering from chronic pain are frequently construed as untrustworthy, manipulative, or engaged in “pain games” (Crowley-Matoka and True, 2012), strategically navigating interactions to secure attention, legitimacy, and secondary gain. Marja-Liisa Honkasalo has likewise documented that patients with chronic pain are routinely dismissed as exaggerating or fabricating their symptoms (Honkasalo, 2001). For our participants, doubt operated not only as a social judgment but also impacted their inner discourse, causing them to question whether it was appropriate or warranted to share their pain with others, whether their experience of pain was “normal”, and even whether their pain was real in the first place. Such findings add important clarity and detail to the still inchoate literature examining the psychosocial dimensions of pain.

Kenneth Craig's widely cited social communication model of pain, building on earlier control experiments, supports the claim that pain experiences are influenced by one's social environment (Craig, 2015). He posits that facets of interpersonal relationships shape how an individual experiences private states; through social interaction, people develop a sense of what others deem an appropriate response to certain painful stimuli and comport their behavior to this response. The findings from our study illustrate how such interpersonal influences operate in a non-controlled, real-world setting with participants who experience disease-specific chronic pain. Crucially, our results elucidate how power structures enacted in real-world contexts modify individuals’ interpretation of their pain and alter their subsequent behaviors that stem from these socially mediated interpretations. In particular, we found that expressions of doubt caused our participants to question the reality, validity, and clinical significance of their symptoms. They were required to decipher whether their private, first-personal experiences were the equivalent to those of everyone else as well as to consider whether they were responding to their pain in the expected manner, based on their clinicians’ perceptions of what is “normal”.

It could be said, then, that our participants often did not approach their pain in the declarative sense — “I am in pain” — but rather in the interrogative sense: “Am I in pain? And if so, how bad is it, really?” This interrogative stance, rife with uncertainty, reveals the extent to which participants were required to align their inner experience with the normative assumptions of their clinicians, illustrating one sociogenic force behind subject formation in chronic pain. For our participants, doubt limited or prevented them from perceiving their pain in its first instance. It stopped them from attending to their symptoms, refusing the “conscious turning toward” their pain that would render it an embodied fact, a sort of learned inversion of traditional somatic modes of attention (Schütz and Wagner, 1975; Csordas, 2002).

### 5.3. Interpersonal shaping of pain testimony

Constantly questioning the reality of their pain caused some participants preemptively to silence themselves. This aligns with a recent meta-synthesis likewise found that “communicative disenfranchisement” (i.e., dismissal or invalidation) routinely results in avoidance of pain disclosure (Hintz, 2023). Our participants expressed hesitancy in describing their symptoms with others despite their awareness that disclosure was clinically valuable – and in fact prerequisite – to receiving appropriate care. Invalidation resulted in their belief that their pain did not warrant sharing. This silencing underscores the sociogeny of pain, by which external doubt is internalized and shapes personal attitudes towards pain. Such invalidation led our participants to believe that they lacked the epistemic authority to interpret their own private experiences.

Doubt was an emotional experience for our participants and diminished their desire to disclose or attend to their pain. Previous studies affirm that experiencing pain invalidation results in feelings of frustration and anger (Linton et al., 2012; Edmond and Keefe, 2015; Vangronsveld and Linton, 2012). Notably, in our study, we found that this trepidation was felt even with *anticipated* invalidation rather than only with *actual* instances of doubt. Additional studies have found that doubt leads patients into isolation and withdrawal from sources of social support, such as friends and family (Hintz, 2023; Clarke and Iphofen, 2005; Dennis et al., 2013; Holloway et al., 2007; Liedberg et al., 2006; Snelgrove and Liossi, 2009). We have reported similar challenges among patients with EDS (Halverson and Colin, 2024). Likewise, a recent systematic review of 151 qualitative studies found that patients with various illnesses—including EDS—often experienced invalidation in healthcare which then resulted in self-doubt, frustration, and anger. Additionally, this review found that experiencing invalidation often resulted in patients withholding or downplaying their symptoms when interacting with their clinicians (Bontempo et al., 2025).

When our participants were willing and able to share their pain with others, they deliberately denied its true extent and severity. This self-denial arose from a worry that their true pain would not be believed by their clinicians, a concern they share with many other chronic pain populations (Nicola et al., 2021). This is similar to the form of epistemic injustice identified by Kristie Dotson as “testimonial smothering”. In such instances, individuals limit disclosures about their experiences to “only content for which [their] audience demonstrates testimonial competence” (Dotson, 2011). As our participants anticipated that clinicians and loved ones would perceive them as lacking the epistemic authority to report on their private experiences, they truncated and limited the descriptions of their pain.

In managing their speech for their clinicians’ sake, our participants revealed an awareness of the social roles available to them in clinical encounters. Previous research has identified that healthcare providers often categorize a patient's pain as either real or unreal in order to determine whether they have a clinical and ethical obligation to treat it (Sullivan and Ferrell, 2005). In our study and others, participants demonstrated a clear awareness of the power asymmetry between them and their clinicians (Halverson and Doyle, 2023; Halverson et al., 2023b). They recognized that they needed to act and speak in a manner alien to their lived experience but necessary for their extraordinary pain to be believed and validated by clinicians. As a result, they fitted their self-reports to these inapt metrics and vocabularies – consciously at first, and then increasingly less so. Over time, the mediation of biomedical language and expectation became internalized, shaping their subjectivity as patients. Thus, they recognized their dependence on clinicians to categorize their pain as real and worthy of attention in order to secure timely diagnosis, a validated sick role, and appropriate treatment.

### 5.4. When pain exists for others

In adjusting their pain testimonies to better align with the expectations of their clinicians, our participants began internalizing these perspectives as accurate interpretations of their subjective experience. Such self-denial aligns with George Herbert Mead's claim that speech enables “the individual to take the attitude of the other towards himself” (Mead et al., 2015). When patients convey their pain through speech, they hear and interpret their own testimony just as much as their intended audience hears and interprets it. For our participants, an anticipation of their clinicians’ dismissal caused them to amend their pain testimony, downplaying and denying its reality and true intensity, thereby taking on a self-conceptualization of their pain as unbelievable. In this way, too, we see that pain experiences are shaped sociogenically by social activity and intersubjective recognition.

When our participants managed to obtain care for pain, they were often required to describe it using the language and metrics of the medical system, alienating them from their own experiences. As Fanon notes, “to speak is to exist absolutely for the other”; language forces speakers to articulate themselves on others’ terms (Fanon, 2002). For example, our participants were often required to assess their pain using the numeric pain scale. However, in a manner consistent with earlier studies on chronic pain, they felt the scale inadequately captured their symptoms, reducing a saliently multidimensional qualitative experience to a unidimensional quantitative value (Halverson and Doyle, 2023; Halverson et al., 2023b; Collins et al., 2022; Robinson-Papp et al., 2015). Much like religious, musical, or dreamlike experiences, anthropologist Jean Jackson argues, chronic pain exceeds the capacities of everyday language (Jackson and Csordas, 1994). However, in order to appear to their clinicians as credible and worthy of treatment, our participants were forced to articulate their internal states through the standards of “normal” pain experiences. As such, these scales required their pain to exist not on its own terms, but rather on the terms set out by their clinicians.

In addition to its descriptive limitations, participants identified a problematic normative force to numeric pain scales: Their inability to account for our participants’ pain led many participants to conceptualize their experiences as unsuited to the standardized tools – and thus, the standardized subject roles – of biomedicine. In struggling to articulate themselves in this manner, they failed to be appropriately “hailed,” in Althusser's terms.

The resulting alienation is analogous to Rosemarie Garland-Thomson's description of misfitting, which theorizes disability as a mismatch between body and environment. “A misfit occurs when the environment does not sustain the shape and function of the body that enters it.” (Garland-Thomson, 2011) Pain scales function as a tool within the institutional environment of medicine, condensing the quality and quantity of a person's pain into a standardized metric that can be inscribed into a medical record and used to guide treatment decisions (Robinson et al., 2024). For our participants, however, these tools rendered them “misfits” within the clinical environment. Moreover, they internalized this misfitting: They revised how they rated their pain, rejecting their own language and evaluations in favor of the ill-suited metrics of the biomedical institution.

Ultimately, our participants, who frequently experienced chronic and severe pain, had to comport their language and modify their behaviors to suit the expectations and worldviews of those who likely have not experienced such debilitating pain. In doing so, they had to imagine the attitudes and beliefs of others and then determine whether their own pain response would align with these attitudes and beliefs. Put more simply, when engaging with others, our participants did not ask themselves: “How would a person who experiences my pain perceive me?” but rather asked themselves, “How would a person without my pain perceive me?” The person-in-pain must envision or imagine the mind of the person-without-pain in order to determine how best to express aspects of their pain experience meaningfully and practically to others (Dennett, 1978; Wimmer and Perner, 1983; Baron-Cohen et al., 1985). Future studies of chronic pain could investigate such a theory of mind as it pertains to pain and pain communication.

### 5.5. The masks of pain

In the state apparatus of biomedicine, clinicians not only manage their patients’ pain but also normalize and legitimize it. For patients living with EDS, this knowledge and power asymmetry compels them to comport themselves in a manner that appeases their clinicians, to take on a language and worldview that often feels alien and alienating. The need to wear “masks” in this way is, of course, one of the central theses of Fanon's *Black Skin, White Masks*. As was described above, our participants “masked” their pain by modifying their language, demeanor, and affect to suit their clinicians’ expectations.

Our participants’ masking was initially intended to signal to their clinicians that their pain was legitimate and worthy of treatment. They strategically comported themselves and amended their speech in a manner that they believed would make a clinician more willing to attend to and accept their pain as medically significant. Such a response is aligned with Linehan's discussion of invalidating environments and how individuals often cope with such environments by becoming compliant with the norms and attitudes expressed in them (Linehan, 1993). This self-denial had a secondary effect: we found that our participants subsequently began to inhabit an attitude of disbelief towards their own sensations, going as far as to question whether their pain actually existed or existed in such a way that was worthy of clinical attention. They not only anticipated dismissal; they internalized it, illustrating the profound – and profoundly embodied – psychosocial consequences of testimonial injustice.

Masking brought about an internalization of doubt similar to the phenomenon of introjection observed in clinical psychotherapy. Through introjection, a patient borrows or mimics their clinician's attitude towards their own physical or emotional injury. Speaking figuratively, the patient wears the “mask” of their clinician within their own self-talk and mimics the stances that they believe their clinician holds toward them. Such introjection can even cause patients to mirror their clinician's dismissive stance (Eekhoff, 2018; Ferenczi and Richard, 1916; Ferenczi et al., 1995). Our participants frequently encountered judgments framing them or their complaints as unreal, unwarranted, or “crazy”. This finding is congruent with similar studies in other populations (Werner et al., 2004; Åsbring and Närvänen, 2002; Shapiro et al., 1997; Shapiro and Teasell, 1998), but we additionally found that our participants internalized this label. They described feeling “crazy” to believe in the reality of their private experiences. This finding also aligns with Beck's discussion of cognitive distortion (Beck, 1980). Namely, participants felt that the invalidation of their symptom reporting was ultimately an invalidation of themselves and their ability to judge reality. Our study suggests that recurrent testimonial injustice can sediment into a self-denying self-conceptualization, shaping how patients living with chronic pain come to live – and embody – the doubt of others.

Our analysis of the sociogeny of pain complicates its conceptualization as “original firsthand experience” (Geniusas, 2020). While it is undeniably true that pain originates in the person who undergoes it, its constitutive features – its normality, dangerousness, and even its perceptibility – depend on social factors outside that person. Hence, while it may be referred to as “private”, its phenomenology is nevertheless shaped by sociogenic forces which inform the “what it is like” characteristics of pain. The pain–rejection feedback loop completes a circuit: Participants doubt pain as unworthy of medical attention, as less severe than they had initially assessed, or perhaps altogether unreal. Masking, thus, does not conclude in a simple, outward performance for others but in a habituated, inward stance toward the self and its first-personal experience.

### 5.6. Limitations, strengths, and future directions

Regarding the study's limitations, our participant sample lacked demographic diversity, with most being white, female, and well educated. Additionally, we relied on self-report to collect data regarding certain clinical features such as diagnosis, such reliance may have introduced some bias or inaccuracy into our data set. It should also be noted that this qualitative data set contains only our participants’ recollection of past events and experiences rather than in-clinic conversations between patients and providers. This limits our ability to assess how the power dynamics that organize a clinical encounter play out in real time.

Concerning the strengths of our study, the participant sample contained individuals from different countries across North America and Europe. This geographic diversity allowed us to capture how provider doubt, as well as its consequences, are potentially international in scope rather than particular to single country's healthcare system(s). Another strength of the study is the inclusion of participants from EDS subtypes diagnosed via genetic testing (i.e., mdEDS) in addition to participants with subtypes diagnosed through clinical criteria alone (i.e., hEDS). This indicates that issues of provider doubt are not insular to a singular subtype of EDS but rather span across the EDS population more generally.

Based on our findings and subsequent discussion, we offer the suggestion for future research to focus on capturing and analyzing interactions between patients and providers as they occur in real time and within a clinical setting. Such data would be crucial to understanding how individuals wear pain masks during clinical encounters and what effect such masking has on these encounters. Our findings also offer potential implications for clinical practice. The sociogenic characteristics of pain uncovered by our study indicate that although pain may be self-evident, how one chooses to express their pain can be influenced by masks constructed in response to interpersonal or social dynamics. This has the potential to cause conflict and frustration in the clinical setting, as many providers assume that patients can express their pain immediately and in a way that reflects how the pain *really* feels. By reinforcing that both the experience and expression of pain is potentially mediated by past personal experience, prior clinical encounters, and other aspects of one's sociality, providers may feel better able to investigate or evaluate their patients’ pain and how to address it clinically.

## 6. Conclusion

Pain is not a purely “private” phenomenon. Through our data on the experience of chronic pain in the Ehlers-Danlos syndromes, we have argued that it is profoundly shaped by sociogenic forces. Its expression is not only given structure by the language and expectations of the apparatus of biomedicine; its phenomenological experience is constituted in significant part through them. Evaluations of pain as normal, valid, and even perceptible are mediated by interpersonal disbelief, clinical instruments that perpetuate feelings of alienation, and the witnessing of others’ “normal” bodies. Drawing on theories of power and embodiment, we have demonstrated how external doubt becomes internalized, leading to self-denial, promoting care avoidance, and shaping first-personal experience. It is necessary, then, to consider how asymmetries in epistemic authority in the patient–provider relationship produce “painful subjects”: patient who amend their behavior and self-talk in order to appease prevailing clinical attitude towards pain and who, ultimately, come to perceive their pain in these experience-distant and inapt terms. Pain, then, is not experienced merely as an immediate physiological sensation, but as a phenomenon shaped by social and discursive forces.

Work remains in describing pain's sociogenic characteristics in other patient populations. In particular, it is unknown how aspects of oppression experienced by minoritized populations affect self-perceptions of pain within these populations and how such self-perceptions impact healthcare utilization, cause injuries to one's self-esteem, and influence one's overall sense of self. Additionally, it is unclear how sociogenic aspects of pain compare between different patient populations. For example, it is unclear if the sociogenic processing of common pain-associated conditions (e.g., cancer) share similarities and differences with the sociogenic processing of pain conditions facing high levels of symptom dismissal or contestation (e.g., fibromyalgia). We invite future investigators to study and address these knowledge gaps.

## Figures and Tables

**Table 1:** Summary of key findings.

Major Theme	Subtheme	Summary
Professing Pain	Pain-Rejection Feedback Loop	Clinician dismissal reduced participants’ willingness to report this pain, which led to inadequate pain management and the eventual worsening of symptoms. As pain or symptoms became unbearable, our participants were forced to seek care, where they once again faced clinician dismissal. The cyclical nature of this dismissal is captured in a pain-rejection feedback loop.
	Anticipating Dismissal	Participants often anticipated that their clinicians would dismiss their pain. This led them to preemptively minimize or alter how they expressed their pain. In some cases, participants felt that they needed to adapt to clinical norms or expectations about pain in order to have their pain taken seriously.
	Clinical Relevance of Pain	Since participants saw disclosing their pain as having negative clinical consequences, they often questioned whether their pain was significant enough to share. Some participants expressed that they only shared their pain if they determined it was worth the potential risk of facing further dismissal by their clinicians.
Normality and Pain	The “Normal” Other	Participants often perceived their pain as normal or unremarkable until they encountered someone that they believed experienced pain “normally”. This led them to alter their perceptions of their pain not through self-reflection but rather through social comparison
	Discovering Abnormality	In certain instances, participants believed their pain was unremarkable until others told them that their pain was abnormal. This assertion of abnormality by others caused our participants to reinterpret their pain experiences through these external perspectives.
	Abnormality in a “Normal” World	Participants interacted with a medical system designed around capturing typical pain experiences through pain scales and other measures. The inability of these scales to capture the atypicality of our participants pain often made them aware of the abnormality of their pain.
The SelfAssessment of Pain	Is the Pain Real	In response to clinician skepticism, many participants began to question the reality of their own pain. This led to feelings that they were imagining or exaggerating their symptoms. Over time, this lead to the internalization of doubt, causing participants to distrust their experiences even in the absence of external dismissal.
	Is the Pain Really That Bad	Participants internalized others’ minimization of their pain which led them to downplay the severity and importance of their pain. Some participants reported that this downplaying caused them to dismiss serious symptoms or push themselves to unsafe limits.
	The Validity of Seeking Care	Due to repeated clinician doubt, participants struggled to determine whether their pain was legitimate enough to seek care. This led some participants to avoid treatment out of fear that they might be perceived as overreacting or wasting a clinician's time.

**Table 2:** Participant information.

Demographic/EDS variable	Interviews (N = 39), n (%)
**Gender**	
Female	29 (74)
Male	6 (15)
Nonbinary	3 (8)
Transgender	1 (3)
**EDS Type**	
Hypermobile (hEDS)	21 (54)
Molecularly defined types (mdEDS)	18 (46)
Classical	7 (18)
Classical-like	2 (5)
Arthrochalasia	2 (5)
Vascular	5 (13)
**Religion**	
Christian	8 (21)
Catholic	10 (26)
Spiritual	4 (10)
None	10 (26)
Other	7 (18)
**Education**	
High school	3 (8)
Some college	7 (18)
Bachelor's degree	13 (33)
Graduate degree	16 (41)
**Race or Ethnicity**	
Non-Hispanic White	33 (85)
Other	6 (15)
**Age (years)**	
Average	42
Range	21–68
